# Nationwide Inequities in Nursing Home Palliative Care Services

**DOI:** 10.1093/geroni/igab046.1274

**Published:** 2021-12-17

**Authors:** Leah Estrada, Jordan Harrison, Andrew Dick, Patricia Stone

**Affiliations:** 1 Columbia University School of Nursing, New York, New York, United States; 2 RAND Corporation, Pittsburgh, Pennsylvania, United States; 3 Rand Corporation, Boston, Massachusetts, United States

## Abstract

Inequities exist in nursing home (NH) quality of care for racial/ethnic minorities, but the extent of palliative care (PC) disparities in unknown. We used cross-sectional national survey data (2017-18) from 869 NHs to measure PC services (summative score: 0-100). Survey linked to Minimum Data Set and Area Health Resources Files. Descriptive statistics and NH-level, multivariable regressions examined regional differences in NH PC services by varying concentrations of Black and Latino residents. Substantial regional differences were recorded in mean PC score and by concentration. Mean PC services were highest in the Northeast and lowest in the South: Northeast (× ®=50.45, SE=1.50); West (× ®=49.96, SE=1.74); Midwest (× ®=48.18, SE=1.17); South (× ®=44.71, SE=1.30). After adjusting for urbanicity and county level poverty, NHs in the Northeast and West with increasing concentrations of Black and Hispanic residents offered significantly fewer PC services. Overall, NHs serving predominantly serving minority populations offer fewer PC services.

